# Modifications of longitudinally extensive transverse myelitis and brainstem lesions in the course of neuromyelitis optica (NMO): a population-based, descriptive study

**DOI:** 10.1186/1471-2377-13-33

**Published:** 2013-04-08

**Authors:** Nasrin Asgari, Hanne Pernille Bro Skejoe, Soeren Thue Lillevang, Troels Steenstrup, Egon Stenager, Kirsten Ohm Kyvik

**Affiliations:** 1 The Multiple Sclerosis Clinic of Southern Jutland (Vejle, Sonderborg, Esbjerg), Jutland Vejle Hospital, Vejle, DK-7100, Denmark; 2 Radiology Clinic, Aleris-Hamlet Hospital, Copenhagen, Denmark; 3 Department of Clinical Immunology, Odense University Hospital, Odense, Denmark; 4 Department of Biostatistics, University of Southern Denmark, Odense, Denmark; 5 Institute of Regional Research, University of Southern Denmark, Odense, Denmark; 6 Institute of Molecular Medicine, University of Southern Denmark, J.B. Winsloewsvej 25,2, Odense C, DK-5000, Denmark

**Keywords:** Neuromyelitis optica, Brainstem lesions, Area postrema, Longitudinally extensive transverse myelitis, Anti-aquaporin-4 antibody, Magnetic resonance imaging

## Abstract

**Background:**

Neuromyelitis optica (NMO) includes transverse myelitis, optic neuritis and brain lesions. Recent studies have indicated that the brainstem is an important site of attack in NMO. Longitudinally extensive transverse myelitis (LETM) is an important component of the clinical diagnosis of NMO. The frequency of brainstem and LETM lesions, changes over time of LETM and the clinical consequences in the course of NMO have only been sparsely studied.

**Methods:**

The study was a population-based retrospective case series with clinical and magnetic resonance imaging (MRI) follow-up of 35 patients with definite NMO and a relapsing-remitting course.

**Results:**

Brainstem lesions were observed in 25 patients, 18 in medulla oblongata (11 in area postrema). Lesions in the pons, mesencephalon and diencephalon occurred in 10, 7 and 7 patients, respectively. Lesions were symptomatic in medulla oblongata and pons, asymptomatic in mesencephalon and diencephalon. Brainstem lesions were observed significantly more often in anti-aquaporin-4 (AQP-4) antibody positive than in seronegative patients (p < 0.002).

LETM was demonstrated by MRI of the spinal cord in 30/36 patients, 23/30 of whom had follow-up MRI of the spinal cord. Recurrent LETM was observed in five patients. In nine patients the LETM changed into multiple lesions during remission or treatment. Spinal cord atrophy was observed in 12/23 (52%) patients, correlating to Expanded Disability Status Scale (r = 0.88, p < 0.001).

**Conclusions:**

NMO patients had frequent occurrence of brainstem lesions and LETM. Brainstem lesions were associated with anti-AQP4 antibody positivity. LETM lesions differentiated over time and the outcome included relapses, fragmentation and atrophy. Correlation was observed between spinal cord atrophy and neurological disability.

## Background

Neuromyelitis optica (NMO) is characterized by inflammation of the optic nerve and the spinal cord [1]. Discovery of serum immunoglobulin G autoantibody towards the water channel aquaporin 4 (AQP4) led to the recognition of NMO patients with clinical signs and/or lesions in the CNS outside of the optic nerve and spinal cord [2-4].

NMO is diagnosed by the demonstration of a combination of clinical manifestations, radiological abnormalities and serological demonstration of anti-AQP4 antibodies [4]. The diagnosis of definite NMO may be made solely on clinical and magnetic resonance imaging (MRI)-based analysis in a high proportion of cases [2,4,5]. However, the demonstration of anti-AQP4 antibodies/NMO-IgG is obligatory in the diagnosis of the NMO spectrum disease, which includes patients with clinical signs and/or MRI lesions in the CNS outside of the optic nerve and spinal cord [4]. Thus, NMO may include more complex and heterogeneous clinical presentations with brain syndromes occasionally leading to considerable diagnostic difficulty. A number of studies have shown brain abnormalities as detected by MRI in 60-71% of NMO patients [3,6-9]. The brain lesions are often localized at sites of high AQP4 expression [10]. The heterogeneous clinical presentations in such NMO patients include brain syndromes such as endocrinopathies [11], posterior reversible encephalopathy syndrome [12] and brainstem syndrome. The brainstem syndrome may lead to respiratory failure [4] or persistent intractable hiccups and nausea [13,14].

Peripheral blood is a likely source for antibody in the CNS [15], but it is not known how anti-AQP4 antibodies reach the CNS [16]. The clinical occurrence of brainstem lesions including area postrema may be related to the areas with high density of AQP4 expression and lack of blood brain barrier [17,18] and it has been suggested that area postrema is a portal of entry to the CNS for anti-AQP4 antibodies [13,18]. However, more detailed studies are required to obtain evidence for the frequency and clinical consequences of brainstem lesions.

In the spinal cord the longitudinally extensive transverse myelitis (LETM) lesion, regarded as typical for NMO, is characterized by involvement of three or more vertebral segments [4]. The changes over time of LETMs and their long term clinical consequences have only been sparsely reported.

The aims of the present study were to estimate the frequency of abnormalities of the brainstem and the spinal cord lesions during the course of NMO and to obtain information about dynamic changes of spinal cord lesions during long-term follow-up. Symptoms and clinical findings were reported.

## Methods

### Study design

A clinical database for NMO patients diagnosed in the time period 1998-2008 in the Region of Southern Denmark was established as part of a population-based study reported in detail elsewhere [2]. The study was a population-based retrospective case series with longitudinal prospective follow-up as described in detail previously [2]. NMO patients were diagnosed according to the Wingerchuk 2006 criteria [4]. Information was obtained by means of review of medical records, a questionnaire, a clinical examination, re-evaluation of previous MRIs of CNS, study examination of supplementary MRIs and serum anti-AQP4-antibody determinations.

### Clinical material

A total of 36 patients with definite NMO were identified in the database and included in the present study. All patients were Caucasians except one. All had a relapsing-remitting course except one, who had a monophasic course. The female: male ratio was 2.8: 1 and mean age at onset was 35.6 years (15–64 years). Disability was retrieved from the medical records where it had been measured by Expanded Disability Status Scale (EDSS) [19].

### Radiographic material

Since the study was retrospective, several types of MRI scanners were used with a variety of imaging techniques. T2-weighted (T2W), T1-weighted (T1W) images with or without gadolinium (Gd), diffusion-weighted imaging (DWI) and fluid-attenuated inversion recovery (FLAIR) sequences were analysed in MRIs of brain. T2W, T1W with or without gadolinium and short tau inversion recovery (STIR) sequences were analysed in spinal cord imaging. Supplementary MRIs of CNS were performed on a 1.5 Tesla scanner (GE, Paris, France). The typology and characteristics of brainstem lesions were described in detail based on a combination of information from the axial and/or sagittal T2-weighted, the T1-weighted + gadolinium and FLAIR MRIs. The lesions (≥ 3 mm) were described by location (infratentorial, medulla oblongata, area postrema, pons, mesencephalon, diencephalon). Spinal cord MRI was either reported as normal, as abnormal with a shorter lesion not suggestive of NMO, or as LETM (cord lesion extending 3 or more vertebral segments). LETMs had a high signal on T2-weighted images and if obtained during acute episodes of myelitis showed hypointensity on T1-weighted images. Spinal cord atrophy was defined as a sagittal diameter of ≤ 4 mm on T1 weighted sequences [20] and was classified with regard to extent of atrophy, 0 denotes no atrophy, focal denotes atrophy of limited extent, and general atrophy denotes changes involving both cervical and thoracic spinal cord. The neuroradiologist was blinded to clinical history and results of other investigations. MRI data were reported in a written.

### Anti-AQP4 antibodies

IgG AQP4 antibodies were measured with a recombinant immuno-fluorescence assay using HEK293 cells transfected with recombinant human full-length AQP4 gene [21,22]. Materials were obtained from Euroimmun (Lubeck, Germany). Patient sera were screened at a 1:10 dilution. Analyses were done in an accredited laboratory at the Department of Clinical Immunology, Odense University Hospital.

### Statistical analysis

Spinal cord atrophy was classified as no atrophy, focal atrophy or general atrophy and EDSS was divided in three groups (categorical variables with 3 categories). Intergroup differences were analyzed using polychoric correlation and Fisher’s exact test on the corresponding 3x3 tables. Statistical analyses were performed using Stata 11 (StataCorp LP, College Station, Texas, USA). The limit of significance was chosen as p < 0.05.

### Protocol approvals, registrations, and patient consent

The study which formed the basis for the database was approved by The Committee on Biomedical Research Ethics for the Region of Southern Denmark (Ref. no. S-20080142) and The Danish Data Protection Agency (Ref. no. 2008-41-2826). All patients provided written informed consent.

## Results

### Radiological and clinical characteristics of brainstem lesions

**Figure 1:**
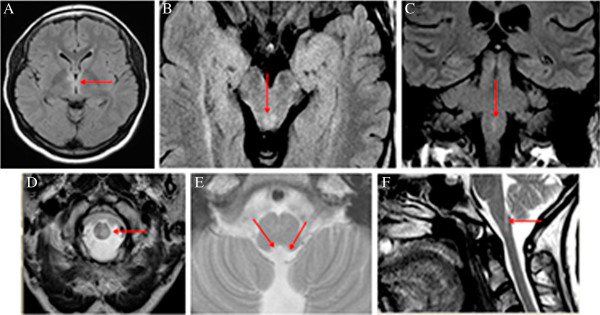
**Typical brain MRI lesions in neuromyelitis optica.** Representative MRI of six NMO patients; Upper row: FLAIR; lower row: T2W, **A**. Lesions in hypothalamic region, **B**. Lesions in periaqueductal matter in mesencephalon, **C** and **D**. Lesions in medulla oblongata, **E** and **F**. Lesions in area postrema of the medulla oblongata.

The frequency of brainstem abnormalities was estimated for 31/35 NMO patients who had available follow-up MRI analysis. Lesions were observed in the brainstem at least once in 25/31 (81%) patients, 18 (72%) of whom were seropositive (Table 1). MRI-lesions in the medulla oblongata were detected in 18 (58%) patients. Of those patients 11 had lesions in the area postrema (Figure 1). Lesions in the pons occurred in 10/25 (40%) and in the mesencephalon in 7/25 (28%) patients. Lastly, hypothalamic and thalamic lesions were observed in 7/25 (28%). A significantly higher frequency of brainstem lesions were observed in anti-AQP-4 antibody positive (18/18) than in seronegative (7/13) patients (p < 0.002).

**Table 1:** Characteristics of brainstems lesions in NMO patients (31)

**Clinical manifestations**	**Anti-AQP4 antibodies positive**						**Anti-AQP4 antibodies negative**					
	**Number of patients = 18**						**Number of patients = 13**					
*MRI of brain*	At disease onset			At follow-up			At disease onset			At follow-up		
Normal	8			2			6			1		
Non-specific changes	10			8			8			6		
MS-like changes*	0			8			0			7		
Topological distribution of Brainstem lesions**	F/m	Sym	Asym	EDDS			F/m	Sym	Asym	EDDS		
				2-4	5-7	8-9				2-4	5-7	8-9
Medulla oblongata	10/2	12	0	4	4	4	4/2	6	0	2	4	0
Area postrema	6/2	8	0	3	3	2	1/2	3	0	0	1	2
Pons	6/1	7	0	2	2	3	2/1	3	0	0	1	2
Mesencephalon	2/1	0	3	0	2	1	3/1	0	2	0	3	1
Hypothalamic and thalamic	5/0	0	5	1	2	2	1/1	0	0	1	1	0

* = meeting the Barkhof criteria for dissemination in space used in the McDonald criteria as described previously [2]. ** = Lesions were observed in the brainstem a total of 25/31 NMO patients.

Based on information from questionnaires and patient files, lesions were uniformly symptomatic in medulla oblongata and pons. Patients with lesions in mesencephalon and diencephalon did not show apparent symptoms. The symptoms were typically polysymptomatic and reversible. The clinical presentation mainly reflected dysfunction of the medulla oblongata and included symptoms such as respiratory failure in 6/31 (19%), 5 seropositive, intractable hiccups and nausea in 9/31 (29%), 8 seropositive, vomiting and nausea in 13/31 (42%), 9 seropositive and bradycardia, blood pressure fluctuations in 5/31 (16%), 4 seropositive. Other clinical signs in the patients with brainstem lesions were: vertigo 23 (74%), 13 seropositive, diplopia 6 (19%), 3 seropositive, facial weakness 2 (6%), 2 seropositive, nystagmus 2 (6%), 1 seropositive and ataxia 4(13%), 3 seropositive. Overall, anti-AQP4-antibody determinations were positive in 72% of the patients.

### Radiological and clinical characteristics of spinal cord lesions

Spinal cord MRI demonstrated LETM in 30/36 patients. Cervical LETM occurred in 21/30 (70%), 8 (27%) reaching into the brainstem. Thoracic TM occurred in 9/30 (30%) cases. Cord lesions involving both cervical and thoracic cord was observed in 13/30 (43%).

The patients with LETM had symptoms including tetraplegia or paraplegia, a well-defined symmetric sensory affection and different degrees of pain and paroxysmal tonic spasms of the trunk and the extremities. The cervical lesions tended to be accompanied by sphincter dysfunction to a higher degree than the thoracic lesions.

**Figure 2:**
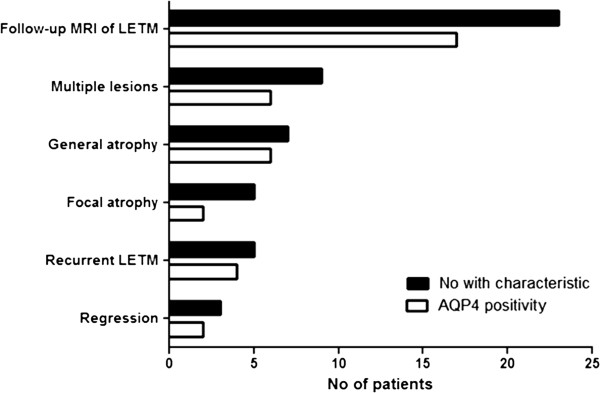
Characteristics of follow-up MRI of longitudinally extensive transverse myelitis (LETM) in 23 NMO patients.

**Figure 3:**
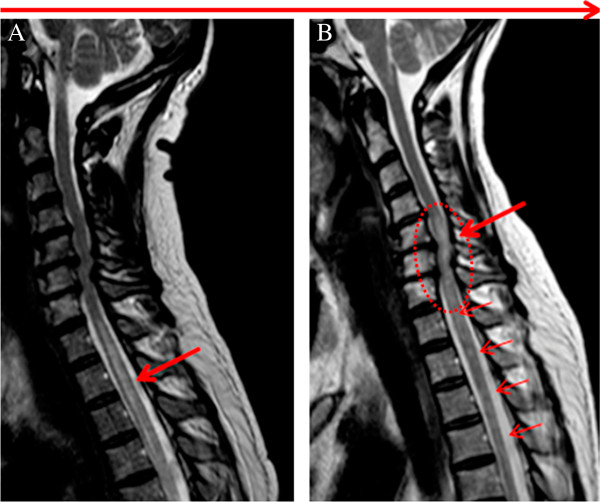
**Modifications of longitudinally extensive transverse myelitis (LETM).** Spinal cord MRI: sagittal T2WI of spinal cord from an anti-AQP4 antibody positive patient with NMO **A**: primary LETM in the upper thoracic cord (arrow) extending from Th1 – 6 (lower limit not shown) **B**: Fragmentation (small arrows) of the earlier LETM following treatment with high-dose steroids and a new LETM (circle) in the lower cervical cord 3 months later.

Out of 30 patients 23 had follow-up MRIs of spinal cord, 17/23 (74%) were anti-AQP4 seropositive (Table 2). Recurrent LETM was observed in 5/23 patients (22%), all female. Following treatment with high-dose steroids, LETMs changed into multiple shorter plaques in 9/23 (39%) patients. Primary MRI was performed within two days of symptoms followed by treatment with high-dose steroids. The time interval from steroid treatment to the control MRI was three to six months (Figures 2 and 3).

**Table 2:** Clinical characterisation and MRI follow-up of NMO patients with longitudinally extensive transverse myelitis (LETM) (n = 23)

**Clinical manifestations**	**F/M**		**Anti-AQP4 antibodies positive**					**F/M**		**Anti-AQP4 antibodies negative**				
			**Number of patients = 17**							**Number of patients = 6**				
			**Duration of disease**		**EDSS Score**					**Duration of disease**		**EDSS Score**		
			**2-4 y**	**5-10 y**	**2-4**	**5-7**	**8-9**			**2-4 y**	**5-10 y**	**2-4**	**5-7**	**8-9**
*MRI of the spinal cord*														
Single LETM	14/3	17	10	7	5	5	7	3/3	6	3	3	1	4	1
Brainstem involvement	7/0	7	5	2	0	1	6	1/0	1	0	1	0	0	1
Relapsing LETM	4/0	4	3	1	0	1	3	1/0	1	1	0	0	1	0
Multiple shorter TM	5/1	6	4	2	0	1	5	1/2	3	2	1	0	2	1
Normal SC	2/1	3	3	0	3	0	0	0	0	0	0	0	0	0
Focal atrophy of SC	0	0	0	0	0	0	0	3/2	5	5	0	0	5	0
General atrophy of SC	4/1	5	4	1	0	0	5	1/1	2	1	1	0	0	2

*F/M* = Female/male, *EDSS* = Expanded Disability Status Scale, *LETM* = longitudinally extensive transverse myelitis, *SC* = Spinal cord, *TM* = Transverse myelitis.

**Figure 4:**
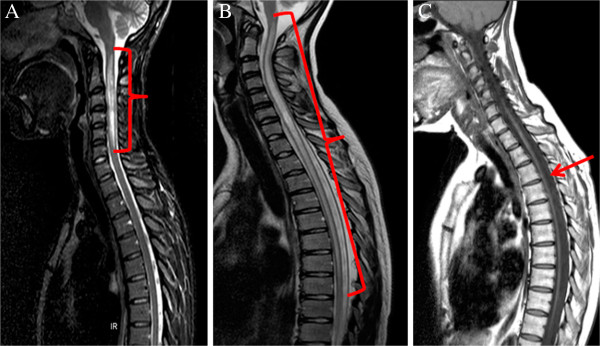
**Longitudinally extensive transverse myelitis (LETM) and atrophy of spinal cord following LETM.** Spinal cord MRI: sagittal T2WI (**A** and **B**) and T1WI (**C**) from three anti-AQP4- antibody positive NMO patients. **A**. MRI showing cervical spinal cord LETM with swelling. **B**. MRI showing LETM of cervical and upper 2/3 thoracic spinal cord. **C**. Severe atrophy of spinal cord as a consequence of recurrent LETM after 6 years duration of disease.

Evaluation of spinal cord atrophy was determined in 23/30 NMO patients who had follow-up MRIs over a period of time. Focal spinal cord atrophy at the site of previous LETM was seen in 5/23 (22%) patients, after 2-4 year duration of disease and with an EDSS score of 5-7. General spinal cord atrophy was observed in 7/23 (30%) patients after 2-4 years duration of disease in two and after 5-10 years in five with an EDDS score of 7-9. A strong correlation was observed (r = 0.88) between the occurrence of spinal cord atrophy and disability as analyzed by the polychoric correlation and the Fisher’s exact test (p < 0.001). Normal appearance of the spinal cord was only observed in 3/23 (13%) patients and myelitis lesions shorter than LETM were found in 7/23 (30%) patients, after 2-4 year duration of disease with an EDSS score of 2-4 (Figures 2 and 4).

## Discussion

In the present study of 35 cases from a population-based NMO cohort a high frequency of brainstem lesions and corresponding clinical signs was observed. Brainstem abnormalities were detected by MRI in 81%, the majority observed in the medulla oblongata (58%) including 35% with lesions in the area postrema. Brainstem lesions were observed more often in AQP4 antibody positive than in seronegative patients (p < 0.002). There was a high degree of agreement between MRI and clinical presentation of brainstem lesions. The study supports the notion that the brainstem, in particular medulla oblongata and area postrema, are important points of attack in NMO [13,18]. These data are in accordance with a multicenter study in Caucasians that found that seropositive patients were predominantly female and had a more severe clinical course [7]. Furthermore, a study from China observed that lesions in the brainstem occurred in a significant proportion of patients [23].

A relative lack of intrathecal synthesis of anti-AQP4 antibodies/NMO-IgG [24,25] and perivascular pathology in NMO suggests entry of antibody from blood vessels to CNS [15]. The BBB restricts entry of serum proteins into the CNS [26]. However, the BBB is not absolute, notably in circumventricular areas including the area postrema [17,18]. Recent studies have suggested that area postrema is a portal for entry of circulating IgG to the CNS in NMO [13,14,18,27].

LETM lesions are regarded as typical for NMO and may be important non-serological markers for NMO [28]. We observed that LETM tended to occur with high frequency during the course of NMO. Furthermore LETM lesions in a considerable proportion of patients changed into multiple fragments during remission or following treatment with high-dose steroids. The present study and other studies [28,29] suggest that evaluation of spinal cord MRI should consider the interval between the acute myelitis attack and the MRI scan. A previous LETM due to NMO may be considered as a differential diagnosis in patients with closely located multiple lesions and atrophy on MRI of the spinal cord. Such an interpretation may help in the understanding of the neurological disability in NMO patients as well as assist in the differentiation of NMO from multiple sclerosis (MS).

It has previously been observed in MS patients that spinal cord atrophy correlates well with concurrent measures of disability [30]. In this study of NMO a strong correlation was observed between spinal cord atrophy and clinical disability as measured by neurological assessment (EDSS). Similar findings of spinal cord atrophy were reported in a study of 20 Afro-Caribbean NMO-patients [20].

Primary strengths of this study were the population-based patient material which originated from a cohort with a high representativity, and the relatively uniform long-term clinical and MRI follow-up. Furthermore, the radiological and the clinical evaluations were performed in a blinded fashion so one specialist did not have the information of the other, in order to minimize bias in the interpretation.

A limitation of this study was the use of different types of MRI scanners with a variety of imaging techniques and a variety of intervals from the onset of clinical manifestations to MRI examination. Also, the clinical evaluation including the EDSS was done by different clinicians at a variety of intervals. However, these limitations reflect the clinical setting of the study as a consequence of its retrospective nature.

## Conclusions

In conclusion the present population-based study demonstrates the frequent occurrence of brainstem lesions and LETMs in NMO patients during the course of NMO. All seropositive patients had brainstem lesions. Modifications of LETMs and brainstem lesions and their relation to the clinical outcome were observed in the course of NMO. The MRI data indicate that LETM lesions differentiated over time to relapses, multiple shorter lesions and atrophy. Consequently, the timing of MRI of the spinal cord may be important for the demonstration of LETM. Large, preferably prospective patient cohort studies are required to obtain more solid evidence on dynamic changes of lesions by MRI and the relationship to clinical presentation as well as to anti-AQP4-antibody levels.

## Abbreviations

AQP4: Aquaporin-4; CNS: Central nervous system; EDSS: Expanded disability status scale; LETM: Longitudinally extensive transverse myelitis; MS: Multiple sclerosis; MRI: Magnetic resonance imaging; NMO: Neuromyelitis optica; ON: Optic neuritis; TM: Transverse myelitis

## Competing interests

Nasrin Asgari has received a travel grant for congress participation from Almirall Nordic. Egon Stenager has received travel grants and support for congress participation from Biogen Idec, Merck Serono, Bayer Schering, Sanofi Aventis and Novartis. The other authors declare that they have no competing interests.

## Authors’ contributions

NA: study concept and design, acquisition of data, and interpretation of results, writing of manuscript. HPBS: MRI re-evaluation and analysis of follow up MRI investigations, revising manuscript and approving final version. STL: Laboratory determination of aquaporin-4 antibodies, revising manuscript and approving final version. TMS: Statistical analysis, revising manuscript and approving final version. ES: Revising manuscript and approving final version, clinical co-supervisor. KOK: Revising manuscript and approving final version, study supervisor. All authors read and approved the final manuscript.

## Pre-publication history

The pre-publication history for this paper can be accessed here:

http://www.biomedcentral.com/1471-2377/13/33/prepub
